# Case Report: DPM1-CDG: Novel Variant with Severe Phenotype and Literature Review

**DOI:** 10.3389/fgene.2022.889829

**Published:** 2022-07-13

**Authors:** Hanna Lausmann, Martin Zacharias, Teresa M. Neuhann, Melanie K. Locher, Karl F. Schettler

**Affiliations:** ^1^ Children’s Hospital St. Marien gGmbH, Landshut, Germany; ^2^ Center of Functional Protein Assemblies, Technical University of Munich, Garching, Germany; ^3^ Medical Genetics Center, Munich, Germany

**Keywords:** DPM1, DPM1-CDG, CDG-Ie, CDG, congenital disorder of glycosylation, epilepsy, neuromuscular disorder, neurodevelopment

## Abstract

**Background:** Congenital disorders of glycosylation (CDG) type I include variants in the *DPM1* gene leading to DPM1-CDG. The nine previously reported patients showed developmental delay, seizures, electroencephalography abnormalities and dysmorphic features with varying disease onset and severity.

**Methods:** Clinical features of a new patient are described. Whole exome sequencing using NGS was performed, followed by molecular simulation of the structural changes in the protein.

**Results:** Our patient with DPM1-CDG presented with more severe symptoms and an earlier onset, specifically non-febrile seizures from the age of 3 weeks, global developmental delay, and severely retarded motor skills. She died at the age of 11 weeks after fulminant sepsis. We identified compound heterozygous variants in the *DPM1* gene, one previously reported point mutation c.1A > C p.? as well as the novel variant c.239_241del p.(Lys80del), resulting in the first in-frame deletion located in exon 2. Loss of Lys80 may lead to an impaired α-helical configuration next to the GDP/GTP binding site.

**Conclusion:** The presented case extends the spectrum of DPM1-CDG to a very young and severely affected child. The deletion of Lys80 in DPM1 results in an impaired helical configuration. This has implications for further understanding the association of structure and function of DPM1.

## 1 Introduction

Congenital disorders of glycosylation (CDG) are a genetically and clinically heterogeneous group of metabolic disorders caused by defects of the glycoprotein and glycolipid glycan synthesis and modification (Sparks et al., 1993; Jaeken and Matthijs, 2001; Grunewald et al., 2002; Grunewald, 2007; Chang et al., 2018). They typically present with a broad clinical spectrum, including neurological abnormalities, facial dysmorphisms, failure to thrive and other organ involvement. The first-line screening for N-linked glycosylation defects is usually performed by carbohydrate deficient transferrin (CDT) analysis (Lefeber et al., 2011). A type 1 serum transferrin isoelectrofocusing pattern corresponds to a CDG-I characterized by defects of dolichol-linked glycan assembly and transfer localized in the cytoplasm or endoplasmic reticulum (ER), whereas a type 2 pattern is associated with a CDG-II characterized by processing defects of protein-bound glycans in the Golgi apparatus.

The activated mannose used for N-glycosylation, glycosyl phosphatidylinositol membrane anchoring, and O-mannosylation of proteins is provided by the glycolipid intermediate dolichol-phosphate mannose (Dol-P-Man). The synthesis of this molecule is catalyzed by the Dol-P-Man synthase complex from guanosine diphosphate (GDP) mannose and dolichol-phosphate (Dol-P) (Maeda et al., 2000). This enzyme is composed of three subunits: dolichol-phosphate mannosyltransferase subunit 1 (DPM1), DPM2 and DPM3 (Maeda et al., 2000). DPM1 is the cytoplasmic catalytic subunit, which is anchored to the ER membrane by DPM3, whereas DPM2 is also localized to the ER membrane and stabilizes the expression of DPM3 (Maeda et al., 2000; Ashida et al., 2006).

Deficiency of Dol-P-Man synthase, caused by variants in the *DPM1* gene, is defined as DPM1-CDG and were first described in 2000 (Imbach et al., 2000; Kim et al., 2000). Nine patients have been reported; they presented with intellectual disability, seizures, microcephaly, and dysmorphic features (Imbach et al., 2000; Kim et al., 2000; Garcia-Silva et al., 2004; Dancourt et al., 2006; Yan g et al., 2013; Bursle et al., 2017).

Here, we describe a female term newborn who presented with a clinically more severe form of DPM1-CDG and died within 11 weeks. She was found compound heterozygous in the *DMP1* gene for a previously described point mutation (Bursle et al., 2017) and for a novel in-frame deletion in exon 2 potentially affecting an α-helical configuration within the DPM1 protein.

## 2 Case Presentation and Methods

### 2.1 Patient Report

We present a female term newborn from a non-consanguineous Caucasian family. She was delivered via Caesarian section at full term after an unremarkable pregnancy. The postnatal course was complicated by apnea and muscular hypotonia, thus she was admitted to neonatal intensive care unit (NICU). She was noted to have dysmorphic signs as rhizomelia, clinodactyly, facial dysmorphia such as high narrow palate, flat nasal bridge, hypertelorism and retrognathia. Her early life was dominated by a global developmental delay with most notably severely impaired motor skills. These were characterized by diffusely reduced muscle tone, incapability of drinking, muted crying and diminished or absent reflexes. She also presented with non-febrile seizures from the age of 3 weeks, characterized by short myoclonic episodes mainly of the limbs, sometimes only seen in electroencephalography (EEG) monitoring without clinical correlate.

An initial laboratory work-up was widely unremarkable, including leucocytes and thrombocytes, basic metabolic panel, infection diagnostics, spinal fluid, creatine kinase (CK) and serum transaminases. Noticeable was merely a mild anemia and a low antithrombin III. A karyotype, testing for Pompe disesase and an extended metabolic work-up (plasma amino acids, urin organic acids, carnitine profile, very long chain fatty acid profile, biotinidase activity) was negative. Ultrasound of the brain revealed pachygyria as well as a dilatation of the left lateral ventricle. An abdominal ultrasound showed enlarged and hyperechogenic kidneys with an enlarged pelvicalyceal system. EEG revealed a burst-suppression pattern with discontinuous background activity, missing sleep-wake cycle and in addition detected multiple short seizures.

### 2.2 Genetic Testing

Whole exome sequencing was carried out on an Illumina NextSeq 500 system (Illumina, San Diego, CA) as 150 bp paired‐end sequencing runs using v2.0 SBS chemistry. Sequencing reads were aligned to the human reference genome (GRCh37/hg19) using BWA (v0.7. 13‐r1126) with standard parameters. Statistics on coverage and sequencing depth on the clinically targeted regions (i.e., RefSeq coding exons and ±5 intronic region) was calculated with a custom script. SNV and INDEL calling on the genes were conducted using SAMtools (v1.3.1) with subsequent coverage and quality dependent filter steps. Variant annotation was performed with snpEff (v4.2) and Alamut‐Batch (v1.4.4). Variants (SNVs/small INDELs) in the coding and flanking intronic regions (±50 bp) were only evaluated.

### 2.3 Structural Modeling and Molecular Simulation

Structural models for both human DPM1 wild type and Lys80 deletion were generated using the Alphafold 2 program (Jumper et al., 2021) with high confidence due to the availability of highly homologous structures from other organisms. The structures were further investigated using Molecular Dynamics (MD) simulations with the Amber18 package (Lee et al., 2018). Both wild type and DPM1 without the Lys80 (Lys80del) were solvated in explicit TIP3P water (Jorgensen and Jenson, 1998) and including Na^+^ and Cl^−^ ions to neutralize the system and adjusting to an ion concentration of 0.1 M. After energy minimization (2,500 steps) and a step wise heating and equilibration the systems were simulated for 100 ns at a temperature of 310 K (37°C) and a pressure of 1 bar.

## 3 Results

**FIGURE 1:**
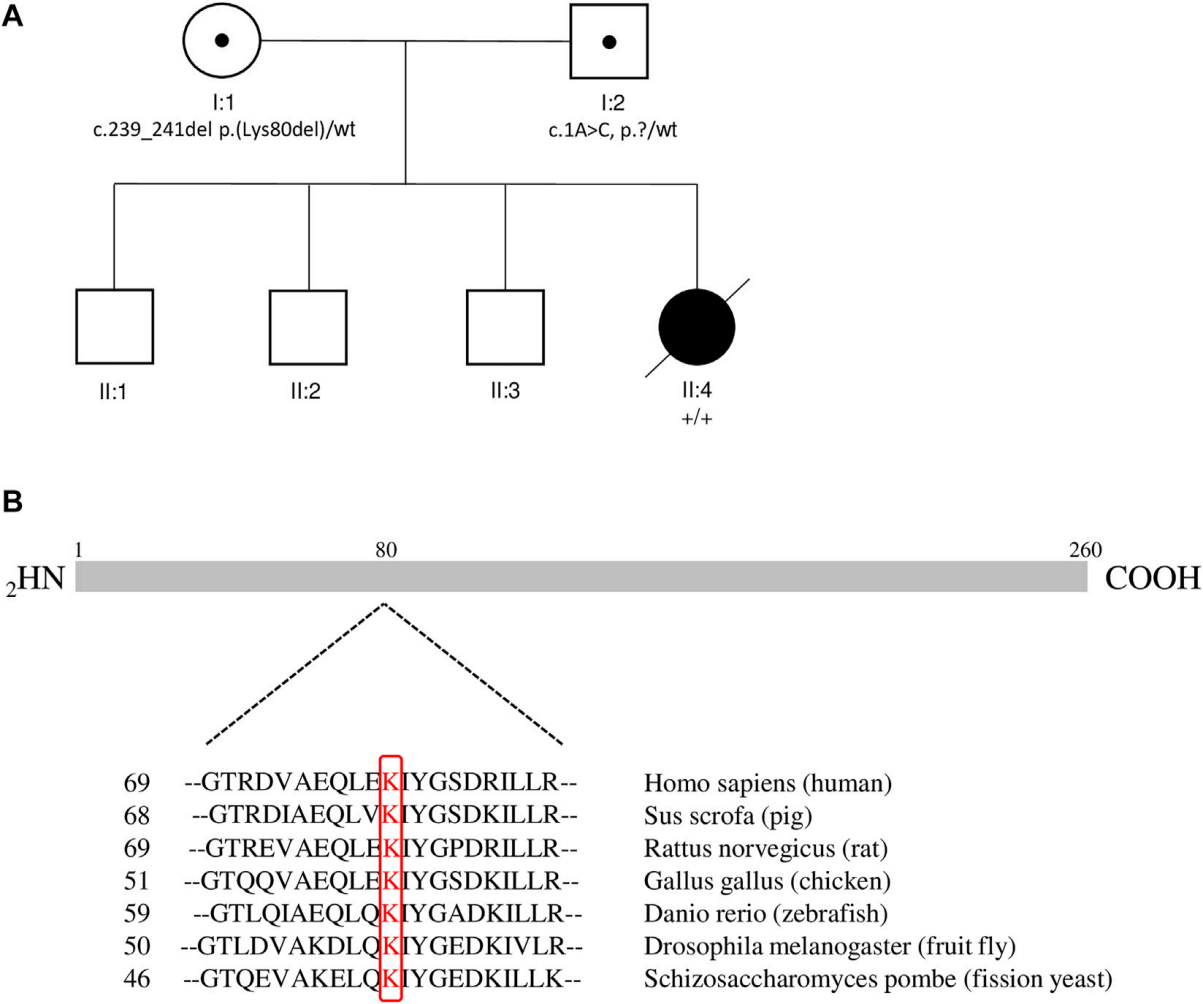
Pedigree and Alignment of Amino Acid Conservation for DPM1. **(A)** The pedigree of the family. Black circle: affected female patient; open squares: unaffected males, open circle: unaffected female; black dots: carrier of the pathogenic variant. **(B)** Alignment of Amino Acid Conservation for DPM1 in Different Species. Sequences are the amino acids for indicated residues of the DPM1 protein in different species. Sequence positions in the respective species are shown on the left. Conserved lysins are highlighted in red. The primary structure of the human DPM1 protein is illustrated in cartoon form above the alignment.

Leading symptoms in the clinical presentation were dysmorphic sign, muscular hypotonia as well as non-febrile seizures while the initial laboratory work-up was widely unremarkable. We additionally performed analysis including karyotyping, testing for Pompe disesase and extended metabolic work-up in order to rule out the differential diagnosis chromosomal defects, Pompe disease and metabolic disorders, respectively. The clinical features as hypotonia and characteristic dysmorphic signs prompted consideration of a CDG. Isoelectric focusing of serum transferrin and alpha-1 antitrypsin revealed a type 1 pattern. We performed whole exome sequencing of the patient (single exome), and in a further step in a segregation analysis using whole exome sequencing of the parents (trio exome) we identified two compound heterozygous sequence variants in the *DPM1* gene, i.e. NM_003,859.2 c.1A > C p.? which was inherited from her father, and NM_003,859.2 c.239_241del p.(Lys80del) which was inherited from her mother (Figure 1A). Variant annotation revealed no other variants compatible with the presented phenotype.

The variant c.1A > C p.? has previously been reported to be linked to DPM1-CDG (CDG-Ie) (Bursle et al., 2017). This variant is predicted to cause a loss of start codon and to interfere with initiation of translation. It is classified as likely pathogenic (Class 4) using the guidelines of the American College of Medical Genetics and Genomics (ACMG, Version 3) (Richards et al., 2015): DPM1 (NM_003,859.2): c.1A > C p.? as Class 4 (PVS1_MOD,PM3,PM2_SUP,PP4).

**FIGURE 2:**
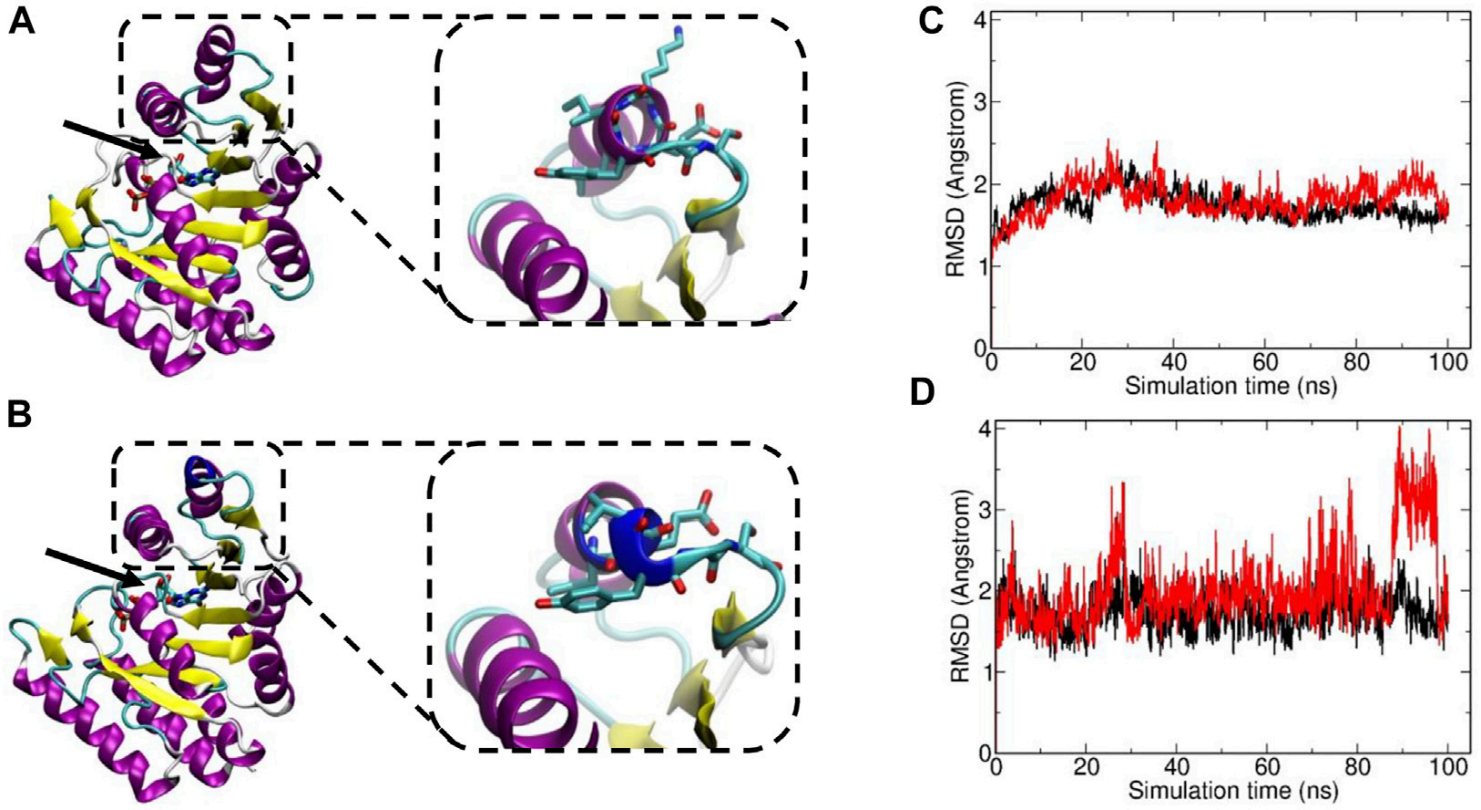
Visualization of Structural Changes of Lys80del in the DPM1 Protein. The DPM1 tertiary structure is shown as color coded cartoon. Alpha helices are colored in pink, beta sheets in yellow, the altered structure near residue 80 in light blue. The location of the GTP/GDP (stick model) is indicated by an arrow. Insets: The location of Lys80 in the one α-helix are highlighted with superimposed amino acids (atom color coded). **(A)** Structure of wildtype DPM1 protein. **(B)** Simulation of DPM1 protein harboring the Lys80 deletion. Dark blue indicates the structural changes in the α-helix. **(C)** Root mean square deviation (RMSD) of the wild type DPM1 (black line) and Lys80 deletion (red line) from the start structure vs. simulation time (ns). **(D)** RMSD vs. simulation time for a segment of ±6 residues around residue 80.

The variant c.239_241del p.(Lys80del) has not been previously described to be associated with DPM1-CDG. It is reported with a low minor allele frequency (MAF) of 4 × 10^−6^ using the population references Genome Aggregation Database (gnomAD) (Karczewski et al., 2020). This likely pathogenic variant leads to an in-frame deletion in exon 2, respectively a loss of residue Lys80. This amino acid is highly conserved in eukaryotes (Figure 1B), demonstrating its fitness advantage and conservation by natural selection. Potential structural changes in the DPM1 protein by the Lys80 deletion were further investigated by structural modeling with Alphafold2 and MD simulations. In the wild type structure Lys80 is solvent exposed and located at one end of an α-helical segment (Figure 2). The structural comparison between the DPM1 wildtype (Figure 2A) and variant (Figure 2B) indicated an intact overall structure with no tendency of global changes or unfolding due to the Lys80 deletion. The root mean square deviation (RMSD) of both proteins from the start structure remained at a relatively constant small level (∼1.8–2 Å, Figure 2C) during the whole simulation. However, the MD simulations revealed a local conformational change and local unwinding of the α-helical configuration near residue 80 (Figure 2B) and also greater fluctuations of the RMSD of this segment (compare red curve of Lys80 deletion and black curve for wild type in Figure 2D). Since this structural element is located in the vicinity of the GDP/GTP binding site in DPM1 the structural changes could potentially affect DPM1 function. In conjunction with the segregation analysis results, this variant can also be classified as likely pathogenic (Class 4) using the guidelines of the American College of Medical Genetics and Genomics (ACMG, Version 3) (Richards et al., 2015): DPM1 (NM_003,859.2): c.239_241del p.(Lys80del) as Class 4 (PM3,PM4,PM2_SUP, PP4).

**FIGURE 3:**
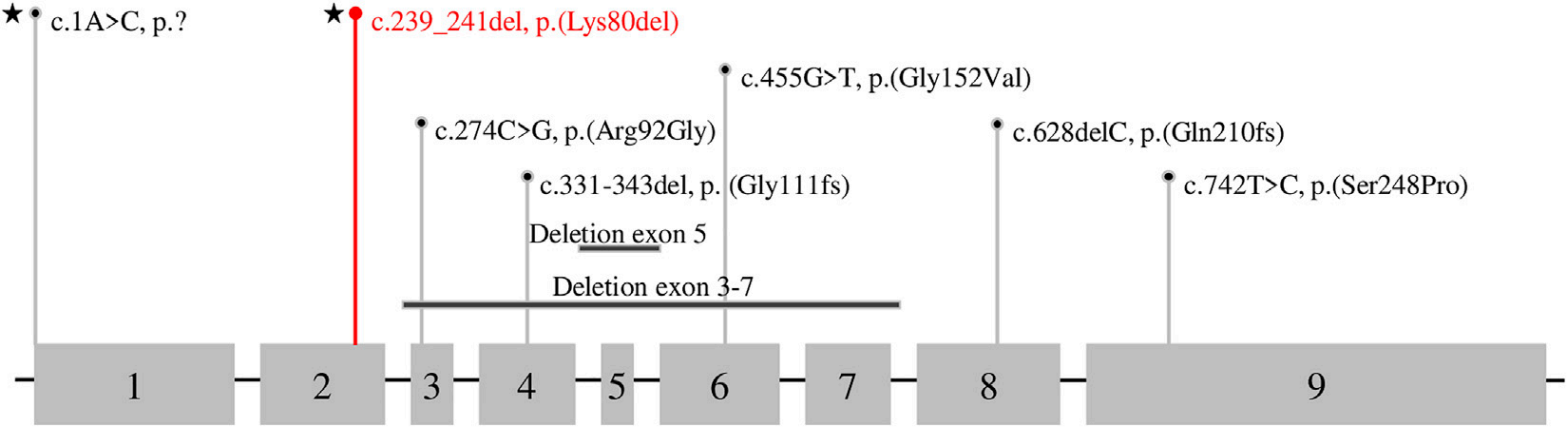
Variant Type and Exon Distribution of Variants in *DPM1*. The *DPM1* (NM_003,859.2, ENST00000371588.5) transcript is shown with numbered exons, grey boxes illustrate the coding sequence. Variants identified in this patient are marked with a star, the novel variant is shown in red and previously reported variants are presented in black.

To date, eight different variants in *DPM1* have been observed to be associated with DPM1-CDG (Figure 3; Tables 1, 2). All previously reported variants are either point mutations or larger intragenic deletions, whereas our variant c.239_241del p.(Lys80del) represents the first in-frame deletion described in DPM1-CDG.

**TABLE 1:** Review of reported DPM1-CDG cases.

Individual	1	2	3	4	5	6	7	8	9	Current Case
age at diagnosis	NA	NA	3 y	19 m	9 y	9 y	7 weeks	9 m	10 y	5 weeks
Gender	female	male	male	female	female	male	female	male	male	female
**variant (NM_003,859.2)**										
cDNA change	274C>G	1: 274C>G	1: 274C>G	1: 274C>G	c.742T>C	373-5T>A	373-5T>A	1: 455G>T	1: 1A>C	1: 1A>C
	Hom	2: 331-343del	2: 628del	2: 628del	Hom	Hom	Hom	2: (261+1_262–1)_(563+1_564–1)del	2: 274C>G	2: 239_241del
amino acid change	Arg92Gly	1: Arg92Gly 2: Gly111 Leufs*45	1: Arg92Gly 2: Gln210 Argfs*4	1: Arg92Gly 2: Gln210 Argfs*4	Ser248Pro	p.?	p.?	1: Gly152Val 2: exon3–7 del	1: Met1Leu 2: Arg92Gly	1: p.? 2: Lys80del
Type of mutation	missense	1: missense	1: missense	1: missense	missense	splice site	splice site	1: missense	1: start loss	1: start loss
		2: frameshift	2: frameshift	2: frameshift				2: deletion	2: missense	2: in-frame del
inheritance	NA	NA	1: mother	1: mother	NA	both parents Het	both parents Het	1: father	1: father	1: father
			2: father	2: father				2: NA	2: mother	2: mother
residual activity of Dol-P-Man synthase	5%	5%	6%	6%	NA	NA	NA	20%	NA	NA
**development**										
DD (+/-)	+	+	+	+	+	+	+	+	+	+
Speech delay (+/-)	NA	+	+	+	NA	+	+	+	+	NA
Motor delay (+/-)	+	+	+	+	+	+	+	+	NA	+
FTT (+/-)	NA	+	+	+	+	+	+	NA	NA	+
**Neurological findings**										
Seizures (+/-)	+	+	+	+	+	+	-	+	+	+
age onset of seizures	10 m	11 m	NA	NA	NA	1y	-	NA	NA	3 weeks
hypotension (+/-)	+	+	+	+	-	+	+	+	NA	+
Ataxia (+/-)	NA	NA	NA	NA	+	+	+	NA	NA	+
**Dysmorphic features**										
facial abnormalities (+/-)	+	-	+	+	+	-	-	NA	+	+
limb abnormalities (+/-)	+	-	+	+	+	-	-	+	+	+
nipple abnormalities (+/-)	-	+	-	-	-	-	-	NA	NA	-
**Additional Findings**										
Microcephaly (+/-)	+	+	+	-	+	+	+	+	+	-
Brain MRI abnormalities (+/-)	-	+	+	+	+	+	+	+	-	NA
EEG abnormalities (+/-)	+	+	+	+	+	NA	NA	+	+	+
CK elevated (+/-)	+	+	+	+	+	-	-	+	+	-
Eye/Vision abnormalities	+	+	+	+	+	+	+	NA	-	+
Clotting System abnormalities (+/-)	+	+	NA	+	+	+	-	+	NA	+
liver function abnormalities (+/-)	NA	+	+	+	-	-	-	+	NA	-
Gastrointestinal disorders (+/-)	NA	NA	+	+	-	NA	+	-	+	-
recurrend infections	NA	NA	NA	+	NA	NA	NA	+	NA	-
CDG Score	NA	NA	NA	NA	NA	NA	NA	15 p (17m), 26 p	NA	43 p (11 weeeeks)
								(32 m), 28 p (4 years)		
**References**	Kim et al. (2000)	Kim et al. (2000)	Imbach et al. (2000)	Imbach et al. (2000)	Garcia-Silva et al. (2004)	Dancourt et al. (2006)	Dancourt et al. (2006)	Yang et al. (2013)	Bursle et al. (2017)	Current

Abbreviations: CK, creatine kinase; DD, developmental delay; Dol-P-Man, dolichol-phosphate mannose; EEG, electroencephalography; fs, frameshift; FTT, failure to thrive; Het, heterozygous; Hom, homozygous; MRI, magnetic resonance imaging; NA, not available.

**TABLE 2:** Review of reported *DPM1* variants.

Variant (NM_003,859.2)	ACMG Criteria (Richards et al., 2015)	Genotype-Phenotype Correlation (ClinVar database)	Amino Acid Conservation	References
c.274C>G	PS4, PS3, PM3, PM2_sup, PP1, PP4	pathogenic	Grantham dist: 125 [0–215]; Comparison between the species: Arg present in 11/13 (total species). Highly conserved amino acid	Kim et al. (2000)
p.(Arg92Gly)				Imbach et al. (2000)
				Bursle et al. (2017)
c.331_343del p.(Gly111Leufs*45)	PVS1, PM3, PM2_sup, PP4	pathogenic		Kim et al. (2000)
c.628del	PVS1, PM3, PM2_sup, PP4	pathogenic		Imbach et al. (2000)
p.(Gln210Argfs*4)				
c.742T>C	PS4, PM3, PM2_sup, PP4	likely-pathogenic	Grantham dist: 74 [0–215]; Comparison between the species: Ser present in 10/13 (total species). Moderately conserved amino acid	Garcia-Silva et al. (2004)
p.(Ser248Pro)				
c.373–5T>A	PM4, PM3, PM2_sup, PP4	likely-pathogenic	Predicted change at acceptor site 5 bps downstream: 49.5%	Dancourt et al. (2006)
p.?				
c.455G>T	PM1, PM3, PM2_sup, PP4	pathogenic	Grantham dist: 109 [0–215]; Comparison between the species: Gly present in 11/13 (total species). Highly conserved amino acid	Yang et al. (2013)
p.(Gly152Val)				
c.(261+1_262–1)_(563+1_564–1)del	PVS1, PM3, PM2_sup, PP4	unknown		Yang et al. (2013)
c.1A>C p.?	PVS1_MOD, PM3, PM2_SUP, PP4	pathogenic/VUS	Change affects the initiator methionine of the DPM1 mRNA. The next in-frame methionine is located at codon 106	Bursle et al. (2017) Current
c.239_241del p.(Lys80del)	PM4, PM3, PM2_SUP, PP4	unknown	highly conserved amino acid (see Figure 1B)	Current

Abbreviations: ClinVar, public database of reports of human variation; VUS, variant of uncertain significance.

At the age of 6 weeks, we were able to make the diagnosis of a DPM1-CDG. However, our patient presented with a progressive neurological and global developmental deterioration including persistent muscular hypotonia, reduced spontaneous movements and seizures. Treatment with multiple anticonvulsants failed to achieve satisfactory control of seizures or improvement of the EEG pattern. At the age of 11 weeks, she showed rare spontaneous movements of her extremities, had no head control nor newborn reflexes. She showed no visual fixation and had feeding problems (exclusive nasogastric tube feeding). Using the Nijmegen pediatric CDG rating scale (Achouitar et al., 2011), we assessed the disease severity at the age of 11 weeks with a score of 43, classified into severe category (>26).

The patient developed a pneumonia resulting in a fulminant sepsis and she died at the age of 11 weeks.

## 4 Discussion

Here we present a child diagnosed with DPM1-CDG, harboring a new variant in the *DPM1* gene resulting in a deletion of Lys80, and showing more severe phenotype than that of previously reported patients.

To date, there are nine cases of DPM1-CDG reported in the literature (Imbach et al., 2000; Kim et al., 2000; Garcia-Silva et al., 2004; Dancourt et al., 2006; Yan g et al., 2013; Bursle et al., 2017), comprising eight different variants in *DPM1* (Figure 3; Tables 1, 2). One of the *DPM1* variants we identified in the patient (c.1A > C p.?) has been previously described and is classified as likely pathogenic. The clinical presentation of the previously described patient with this variant was dominated by seizures, developmental delay, and severe gastrointestinal involvement (Bursle et al., 2017). In this case sequencing revealed two compound heterozygous sequence variants, i.e., c.1A > C p.? also found in the current case, and c.274C > G previously described in four other patients (Imbach et al., 2000; Kim et al., 2000; Bursle et al., 2017).

The *DPM1* variant c.239_241del p.(Lys80del) is predicted to lead to an in-frame deletion of the amino acid lysine in position 80 in exon 2. To our knowledge this is the first in-frame deletion described in DPM1-CDG. Due to the high conservation of this position throughout eukaryotes (Figure 1B) as well as the absence of this variant in a large reference genome of healthy controls, it is likely to be a causative for the severe phenotype. Moreover, modelling of wildtype and variant DPM1 illustrates that the deletion of Lys80 does not strikingly modify the structure of the whole protein, but leads to changes in an α-helical configuration and increased local conformational fluctuations (Figure 2). The affected α-helix is located in the neighborhood of the GDP/GTP binding site, which might indicate a decreased enzyme activity. Note, that the simulations are based on an AlphaFold2-based structural model of DPM1and can only serve to suggest possible explanations for the effect of the mutation. Future experimental studies are required to validate the observed structural changes found in the simulation. Also, substrate binding could be affected, but detailed functional consequences of this single amino acid deletion on the function of DPM1 remains to be analyzed. Thus, we identified the new variant c.239_241del p.(Lys80del) in the *DPM1* gene which appears to be likely pathogenic and possibly causes more severe clinical symptoms in combination with the known likely pathogenic variant.

The clinical features of the reported patient include seizures from infancy, developmental delay, severely retarded motor skills and dysmorphic abnormalities, similar symptoms have been described in previously published DPM1-CDG cases (Table 1). She started having medically intractable seizures from the age of 3 weeks, while onset of seizures in previously described DPM1-CDG patients range from 10 months (Kim et al., 2000) to 1 year or were even absent (Dancourt et al., 2006). However, data availability of seizure onset was limited as only three out of nine previously reported patients state the age of seizure onset. Moreover, she developed severely retarded motor skills including reduced muscle tone and incapability of drinking at the age of 11 weeks. Motor delay as well as muscular hypotension was previously described (Imbach et al., 2000; Kim et al., 2000; Garcia-Silva et al., 2004; Dancourt et al., 2006; Yan g et al., 2013), however not to this extend. As DPM1-CDG has been reported only in nine individuals so far, it is difficult to state an overall prognosis. The clinical course ranges from only mild symptoms (Garcia-Silva et al., 2004; Dancourt et al., 2006) to severely affected individuals with massive physical constraints (Imbach et al., 2000; Kim et al., 2000). The global disease severity can be classified by the previously published Nijmegen pediatric CDG rating scale (Achouitar et al., 2011) into mild (0–14), moderate (15–25) and severe (>26) category. For the reported patient we assessed a score of 43, reflecting a very high level of disease severity. As this rating scale is a recent tool, it was applied only to one previously published DPM1-CDG patient, who scored with 15 points at the age of 17 months and 28 points at the age of 4 years (Yan g et al., 2013). Taken together, the clinical course of our patient seems to be far more severe in comparison to previously reported DPM1-CDG cases.

However, the laboratory workup including serum transaminases and creatine kinase was unremarkable in our patient, other than in previously published DPM1-CDG patients. There are four cases reported to have liver function abnormalities (Imbach et al., 2000; Kim et al., 2000; Yan g et al., 2013). Interestingly, in seven previously published cases creatine kinase was elevated (Imbach et al., 2000; Kim et al., 2000; Garcia-Silva et al., 2004; Yan g et al., 2013; Bursle et al., 2017), while in our case this enzyme was unremarkable. Microcephaly is another clinical feature associated with DPM1-CDG and was observed in eight out of nine cases (Imbach et al., 2000; Kim et al., 2000; Garcia-Silva et al., 2004; Dancourt et al., 2006; Yan g et al., 2013; Bursle et al., 2017), while our patient did not show signs of microcephaly. Yet, her head circumference decreased from birth (35.3 cm, 80th percentile) to the age of 11 weeks (39.0 cm, 60th percentile) and microcephaly might have developed as it is known from one previous case (Imbach et al., 2000).

In conclusion, we present the 10th DPM1-CDG patient, with a severe phenotype and a novel variant, as well as a literature review of this CDG.

## Data Availability

The datasets for this article are not publicly available due to concerns regarding participant/patient anonymity. Requests to access the datasets should be directed to the corresponding author.

## Abbreviations

ACMG, American College of Medical Genetics and Genomics; CDG, congenital disorders of glycosylation; CDT, carbohydrate deficient transferrin; CK, creatine kinase; DD, developmental delay; Dol-P, dolichol-phosphate; Dol-P-Man, dolichol-phosphate mannose; DPM1, dolichol-phosphate mannosyltransferase subunit 1; EEG, electroencephalography; ER, endoplasmic reticulum; FTT, failure to thrive; GDP, guanosine diphosphate; gnomAD, Genome Aggregation Database; Het, heterozygous; Hom, homozygous; MAF, minor allele frequency; MD, Molecular Dynamics; MRI, magnetic resonance imaging; NA, not available; NGS, next‐generation sequencing analysis; NICU, neonatal intensive care unit; RMSD, root mean square deviation.
